# Quantitative Analysis of Droplet Evaporation Based on Wedge Prism Digital Holographic Microscope

**DOI:** 10.3390/mi16101114

**Published:** 2025-09-29

**Authors:** Jiankun Wang, Han Wang, Yang Luo, Zhuoji Liang, Gengliang Chen, Meng Wang, Guoliang Zheng, Xuhui Zhang

**Affiliations:** 1 Sino-German College of Intelligent Manufacturing, Shenzhen Technology University, Shenzhen 518118, China; 2310415029@stumail.sztu.edu.cn (J.W.);; 2 College of Engineering Physics, Shenzhen Technology University, Shenzhen 518118, China

**Keywords:** digital holographic microscopy (DHM), wedge prism, thickness measurement, droplet evaporation

## Abstract

This study presents a prism-based self-referencing digital holographic microscopy (PSDHM) system that utilizes a wedge prism. The front and rear surfaces of the prism have a wedge angle of 2°, which can reflect the parallel incident light, respectively, to generate a lateral displacement that varies with the propagation distance of the optical path. Focusing on the quantitative analysis of droplets, this innovative system effectively images water droplets and their dynamic evaporation processes. Results show that the evaporation process of water droplets undergoes three stages, each stage corresponding to a theoretical model. These are the constant contact radius (CCR) mode, the stick-slip (SS) mode, and the stick-jump (SJ) mode. Furthermore, by comprehensively analyzing the contact angle and the specific morphology of the droplet’s contact area, we revealed that the hydrophilicity of the cover glass influences the droplet morphology, contact area, and the evaporation process.

## 1. Introduction

Digital holographic microscopy (DHM) represents a cutting-edge optical microscopy modality that integrates digital holography, optical microscopy, and image processing techniques. This innovative approach enables the quantitative, rapid, and non-destructive characterization of samples [1,2,3,4,5,6]. Self-referencing configurations are a branch of DHM, where the reference beam is typically derived from the object beam. Both beams traverse identical optical paths and interact with the same set of optical elements. This design effectively mitigates optical path difference (OPD) and enhances the system’s temporal stability, which is crucial for accurately capturing dynamic changes in the samples’ thicknesses [5,6,7,8]. DHM has also been introduced as a practical tool for studying droplet evaporation [9,10,11].

Studies on self-referencing digital holographic microscopes typically focus on the introduction of novel beam-splitting elements to generate interfering beams. For instance, the front and rear surfaces of a glass plate are utilized to form two beams with an OPD [6,7,8,12]. Mirrors [2,13,14] or Fresnel prisms [15,16,17,18,19] are employed to reflect or refract part of the light source to overlap and interfere with another part of the light. A combination of a beam splitter (BS) and double reflectors [5,20], as well as a combination of a polarization beam splitter (PBS), mirrors, and a motorized linear stage [21], are used to realize optical path structures with adjustable OPD. Moreover, filtering in the physical space was used to obtain 0-level and 1-level light and then using them for interference [22,23]. By utilizing the polarization characteristics of PBS, dual-wavelength common-path interference can be achieved using two independent light sources [24]. Even with only one BS, common-path off-axis interference of the light source can still be achieved and the off-axis angle can be adjusted by slightly rotating the BS [25]. Additionally, diffuser-pinhole combined elements [3] and metasurfaces [26] are introduced to achieve interference within specific wavelength ranges or imaging regions.

It is evident that these schemes have more or less shortcomings. For instance, they can only form fixed OPD or lateral displacement and suffer from a small field of view (FOV), limited 3D reconstruction area, difficulty in adjustment, complex post-processing by software, and difficult material preparation.

Traditional optical components, such as thick glass plates [8], can only image samples effectively up to approximately 60 μm. This is because the two beams are nearly parallel, which means the lateral displacement cannot be adjusted. If samples exceed this size, the object beam and reference beam overlap—making 3D topography reconstruction impossible. However, this limitation can be overcome using our PSDHM system. From the experimental results, our PSDHM has successfully captured dynamic images of droplet samples with diameters up to around 400 μm. The system will be presented in detail in Section 2: Methods, and its performance will be verified through droplet experiments, droplet evaporation experiments, and comparisons with other devices in Section 3: Results and Discussion.

## 2. Methods

**Figure 1:**
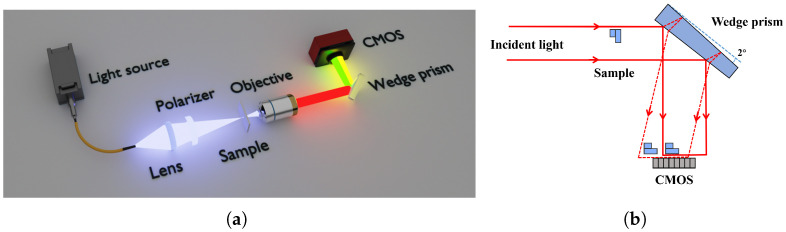
(**a**) Schematic diagram of the optical path structure for the PSDHM. (**b**) The working principle of the wedge prism; the continuous line represents wavefront reflected by the front surface of the prism and the dashed line represents wavefront reflected by the rear surface of the prism.

The experimental setup, as shown in Figure 1a, employs a 450 nm laser source (CC LASER, Changchun, China, MW-SGX-450/50 mW). The emitted light sequentially passes through a lens (JCOPTIX Inc., Nanjing, China, f = 40 mm, OLC240134M) and a polarizer (LBTEK Inc., Changsha, China, FLP25-VIS-M) before illuminating the sample. The transmitted light is magnified by a microscope objective (Olympus, Tokyo, Japan, UPLFLN 4x) and subsequently directed on both front and rear surfaces of the wedge prism (LBTEK Inc., Changsha, China, CWP10), generating two reflected beams with controlled lateral displacement.

The working principle of the wedge prism is shown in Figure 1b; the wavefronts reflected from the front and rear surfaces of the prism result in a noticeable interference phenomenon between the two wavefronts, enabling quite good imaging performance. The CMOS sensor (Thorlabs Inc., Newton, MS, USA, CS165MU/M) will only capture the sample information from the front surface of the wedge prism, while the wavefront of the rear surface serves as a reference.

The interference fringes generated by these two beams can be regarded as a reference beam and an object beam having phase information. The recorded image which has the superimposed state of these two beams on the image sensor is called the hologram $I_{Holo}$, and can it be expressed as follows, where *R* and *O* are the complex amplitudes of the reference and the object beam, and $R^{*}$ means a complex conjugate of *R*, respectively:

(1) $$I_{Holo}={\left|R\right|}^{2}+{\left|O\right|}^{2}+R^{*}O+RO^{*}$$

In the Fourier domain, ${\left|R\right|}^{2}+{\left|O\right|}^{2}$ is the DC spectrum and the $R^{*}O+RO^{*}$ represents the ±1 sidebands. In addition, Equation (1) can be derived as follows, where ϕ is a phase information of the recorded hologram:

(2) $$I_{Holo}={\left|R\right|}^{2}+{\left|O\right|}^{2}+R^{*}Oe^{j\Phi }+RO^{*}e^{-j\Phi }$$

The phase information of the recorded hologram can be obtained from one sideband in the Fourier domain. In this paper, we perform frequency-domain filtering on the +1 sideband. The quantitative phase information can be obtained by calculating the argument as follows, where $\Psi (f_{x},f_{y})$ is the Fourier transform result of $I_{+1}=R^{*}Oe^{j\Phi }$:

(3) $$\phi \left(f_{x},f_{y}\right)=\arg\left[\frac{Im\left[\Psi (f_{x},f_{y})\right]}{Re\left[\Psi (f_{x},f_{y})\right]}\right]$$

At this point, the phase information of the hologram is obtained. Subsequently, phase unwrapping can be performed to retrieve the three-dimensional information of the object contained in the hologram. The three-dimensional reconstruction can be calculated by Equation (4), where Φ denotes the unwrapped phase, λ is the wavelength of the light source (450 nm), and Δn stands for the difference in refractive index between the sample and the surrounding medium.

(4) d=λΦ/2πΔn

## 3. Results and Discussion

### 3.1. Liquid Droplet Experiment

In this experiment, a liquid droplet is placed onto a cover glass using a pipette and directly exposed to air for optical microscopy imaging. A 4× microscope objective with NA = 0.13 was used for magnification. Holograms are recorded on the image plane, and the complex amplitude of the wavefront were obtained through frequency-domain filtering, which allowed us to directly access the phase of the wavefront.

**Figure 2:**
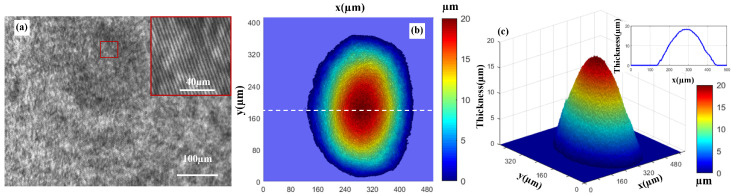
Background hologram and liquid droplet imaging via PSDHM. (**a**) The captured background hologram, insert map shows the enlarged fringe display diagram. (**b**) Thickness distribution of the water droplet. (**c**) 3D rendering of thickness profile in (**b**); the insert map shows the 1D thickness profile along the white dotted line in (**b**).

The captured background image and the enlarged image of the stripes are shown in Figure 2a. Figure 2b records the thickness distribution of a liquid droplet under the camera’s field of view, Figure 2c is a 3D display of Figure 2b, and the insert map shows the cross-sectional thickness distribution of the liquid droplet along the line in Figure 2b.

### 3.2. Droplet Evaporation Experiment

**Figure 3:**
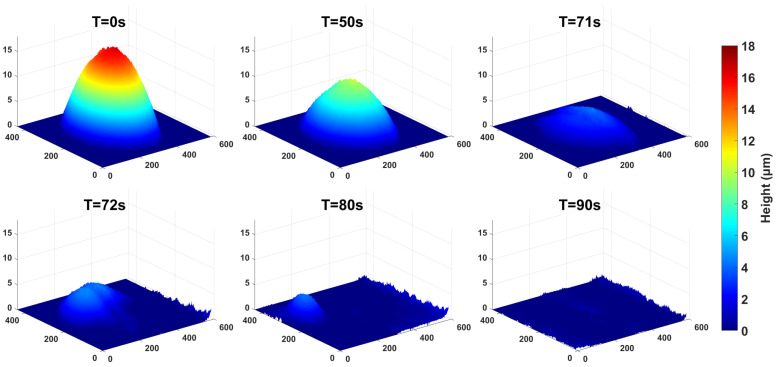
The process of droplet evaporation. The scale units for the x, y, and z coordinates of the subplots are all in μm.

The evaporation experiment is performed at open conditions, with a relative humidity (RH) of 68% and ambient temperature (AT) of 24 °C. The droplet is placed onto a cover glass using a pipette. Set to a 1 s shooting interval, the camera keeps capturing images until the droplet completely evaporates, a process that takes 90 s in total. The dynamic evaporation process is illustrated in Figure 3.

**Figure 4:**
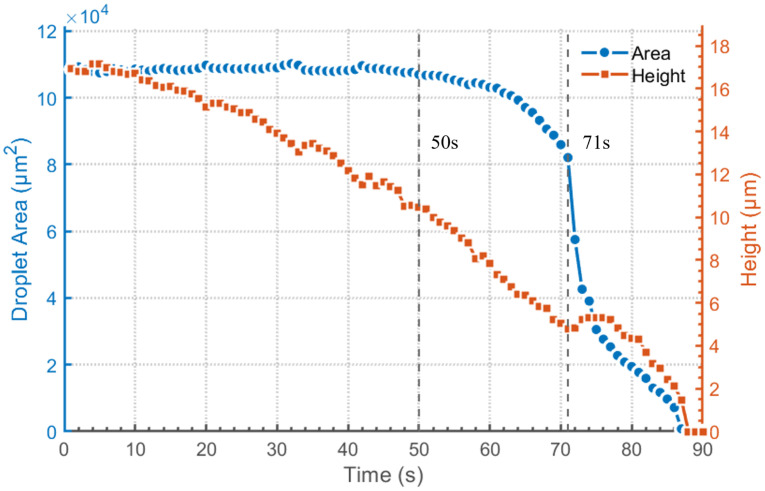
Changes in droplet contact area and height over time.

An analysis is conducted on the changes in the contact area and height of the droplet over the 90 s period. The results are shown in Figure 4. The blue line represents the contact area of the droplet, while the orange line represents the droplet height. From the experimental results, it is evident that the evaporation process of the droplet can be divided into three stages. In the first stage, from 0 to 50 s, the water droplet evaporate in the constant contact radius (CCR) mode, with the height and contact angle decreasing continuously. In the second stage, from 50 to 71 s, both the contact angle and contact radius of the water droplet decrease simultaneously, which is referred to as the stick-slip (SS) mode. In the third stage, the water droplet enter the stick-jump (SJ) mode. In this mode, the retraction speed of the contact line is faster than the decrease rate of the droplet height. This results in a sudden change in the droplet shape after the contact line jumps, leading to a phenomenon where the droplet height increases instead of decreasing and the droplet area shrinks sharply. This is consistent with the droplet changes shown in Figure 3, which occurred around 71 s. We have carried out a series of experiments. The droplet goes through three similar stages, which are CCR, SS, and SJ. Moreover, the time points for switching between different stages are also very close.

**Figure 5:**
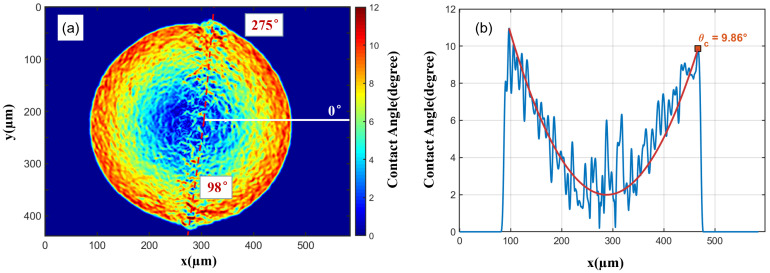
The top view and gradient profile of the water droplet at T = 0 s. (**a**) The top view of the water droplet gradient, the white line denotes the initial 0° position, and the red dashed lines indicate the positions where abrupt changes in the contact angle occur. (**b**) The gradient profile of the water droplet along the 0° direction; the red curve is used to represent the overall gradient variation within the droplet. The unit of the gradient in the figure has been converted to the actual contact angle value using arg.

Subsequently, the analysis and test of the water droplet contact angle are shown in Figure 5. The contact angle can be obtained by calculating the gradient σ of the water droplet height function f(x,y), as described in $\sigma =\sqrt{{\left(\frac{\partial f}{\partial x}\right)}^{2}+{\left(\frac{\partial f}{\partial y}\right)}^{2}}$. This gradient represents the slope variation of the water droplet surface. This is reflected in Figure 5a as high gradient values at the edge of the water droplet, which is due to the abrupt change in slope from the cover glass to the water droplet; meanwhile, the gradient gradually decreases toward the interior of the water droplet. Figure 5b shows a cross-sectional view of Figure 5a along the 0-degree direction. The maximum gradient value $\theta_{c}$ appearing at the water droplet edge indicates the gradient value at the contact radius of the droplet. Thus, it is recorded and converted into the actual angle through $\theta_{c}=\max\left\{\arg(\sigma )\right\}$ to intuitively display the magnitude of the contact angle, while the red line represents a fitted curve to illustrate the gradient variation inside the water droplet.

In Figure 5a, it is not difficult to find that along the two red dashed lines at $98^{\circ }$ and $275^{\circ }$, the edge of the water droplet is not smooth and the sizes of the contact angles are significantly different.

**Figure 6:**
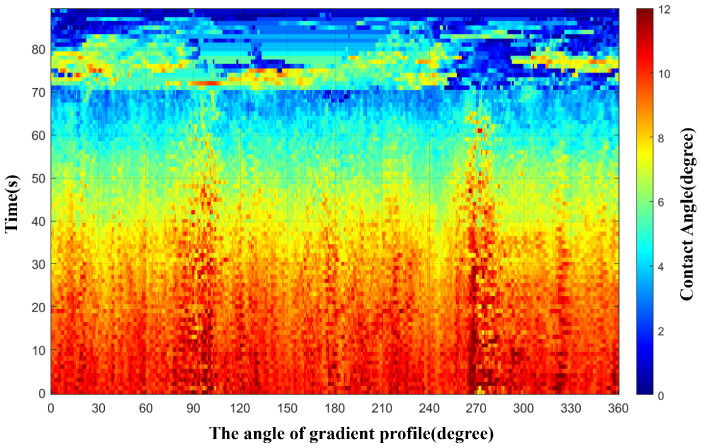
Contact angles in all directions around the droplet perimeter obtained by sectioning at each time point from 0 to 90 s.

In order to further analyze the abrupt change in the size of the contact angle and the variation of this abrupt phenomenon in the time domain, we performed 360-degree gradient sectioning on a single water droplet image and searched for the maximum gradient at the droplet edge to obtain the contact angles in all directions around the droplet perimeter. The results are shown in Figure 6, where all data recorded within 90 s were processed for contact angle analysis. The abscissa represents the variation of contact angles along the 360-degree range of a single image, and the ordinate represents the variation of contact angles at a certain section angle across different time. Two key phenomena are evident from the figure. First, before 71 s, there is an obvious climb in the size of the contact angle at $98^{\circ }$ and $275^{\circ }$. Second, within the first 71 s, the contact angle showed a uniform and gradual decrease overall. After 71 s, due to the morphological abrupt change and displacement of the water droplet, the contact angle exhibited obvious discontinuities, the values increased instead, and the abrupt change in the contact angle has disappeared, which is consistent with the intuitive observation of the dynamic evaporation process in Figure 3.

**Figure 7:**
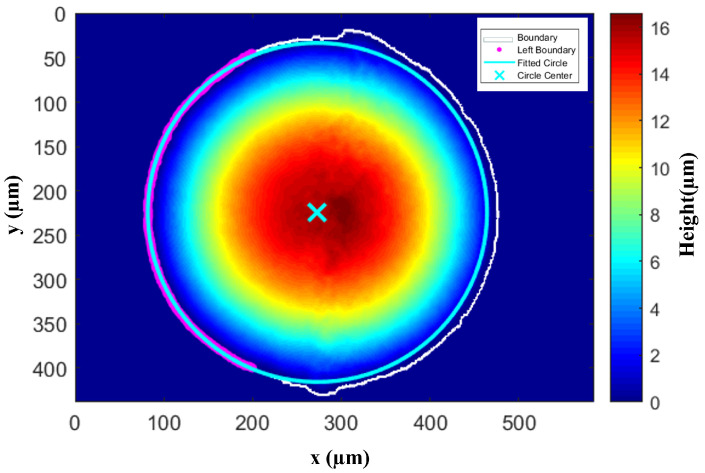
Top view of the water droplet height at T = 0 s. The purple arc represents the droplet edge data used for fitting a complete circle, the blue circle denotes the fitted complete circle, and the cross in the middle indicates the center of the fitted circle. The white line shows the real distribution of the droplet edge.

We also researched and analyzed the edge morphology of the water droplet. As shown in Figure 7, which displays the top view of the water droplet height at T = 0 s, we selected the boundary data (purple) of the droplet to the left of x=200μm to complete circular fitting (blue), with the cross in the middle denoting the center of the fitted circle. The plotting results show that the water droplet was not a regular circle. On the right side of the droplet, the contact area exceeded the range of the fitted circle, and the starting boundary of the excess part was precisely near $98^{\circ }$ and $275^{\circ }$, as mentioned in the above phenomenon. The reasonable explanations for this phenomenon are as follows.

First of all, the water droplet is not vertically dropped onto the cover glass but formed on the cover glass surface through oblique incidence by a pipette. Therefore, the initial morphology of the water droplet’s contact area is not a regular circle. Moreover, the water droplet is formed on the cover glass, and the siloxane bonds (Si-O-Si) on the cover glass surface readily form hydrogen bonds with water molecules. This chemical interaction endows it with natural hydrophilicity, which enhances the water droplet’s ability to maintain its initial morphology after oblique incidence. Therefore, this phenomenon was well preserved within the first 71 s.

Additionally, this phenomenon changes and disappears rapidly after 71 s of droplet evaporation. As shown in Figure 3, the sudden change in contact angles at $98^{\circ }$ and $275^{\circ }$ dissipates after 71 s, and the contact angle values of the water droplets at the same time are in a state of concentrated distribution. The reason is that after 71 s, the water droplet is in the SJ mode, where the surface tension of the water droplet can no longer maintain its original shape and thus collapses rapidly. That is, the contact area of the water droplet shrinks or even shifts as a whole, as shown in the subplots of T = 71 s and T = 72 s in Figure 3. This process occurs quickly, which is reflected in Figure 6 as an obvious discontinuity in the change of the water droplet contact angle at 71 s.

There are uncertainties in the experiment, and the analysis is as follows. First, AT and RH affect the evaporation process of the droplet. We should try our best to keep the AT and RH consistent during the experiment. Second, there is the influence of light in the open experimental environment. Changes in ambient light will lead to discontinuous droplet evaporation during dynamic shooting and noise in a single photo. This noise will be amplified in the gradient map. Third, there is the influence of dirt and dust on optical components. The phase information of the light source will accurately record the influence of such dirt and dust on the optical path, which is specifically reflected in the inherent background height during 3D reconstruction.

The inherent background influence caused by dirt on optical components in the experiment can be well solved through the post-processing method of background subtraction. However, in other aspects, based on the current conditions of the laboratory, we cannot accurately adjust the AT and RH or completely isolate the influence of ambient light. This constitutes a limitation of the experiment. The influence of AT and RH on droplet evaporation is worthy of in-depth research.

### 3.3. Device Performance and Comparison

The PSDHM is built on a 150 × 300 mm breadboard using a coaxial structure. Except for the wedge prism that acts as the core of the structure, all other components can be replaced. This makes the device compact and easy to reproduce. To show the device’s performance better, we conduct a resolution test. We use a USAF 1951 standard resolution chart for the test. The test results show that the smallest distinguishable horizontal unit is Group 7, Element 2. The line width of one unit is 2.76 μm.

At the same time, we also make a comparison table for horizontal devices. The size of the field of view and the fringe ratio within the FOV are also important. They comprehensively reflect the device’s practical usable FOV and the maximum size of samples that can be imaged. We have also listed these in Table 1.

## 4. Conclusions

In this paper, a wedge prism is introduced into DHM to realize compact-structured holographic imaging. PSDHM represents a compact, portable, and practical novel common-path off-axis DHM configuration. We conducted an experiment on forming a single liquid droplet, which has a diameter of approximately 400 μm. We first shot the process and then reconstructed it. The results show this method works well. It can clearly image the large-sized droplet and dynamically record how the droplet evaporates.

During the droplet evaporation experiment, the process initially follows the CCR mode and then transitions to shrinkage via the SS and SJ modes. This experimental result is in line with the liquid droplet evaporation model summarized by experts in the field of water droplets. Moreover, the time points for switching between different stages are also very close. This phenomenon is very interesting, but we have limited knowledge of the relevant theories of droplet evaporation at present. We cannot explain it clearly for the time being. We will continue to conduct relevant research around this topic.

In the future, we will apply for a dedicated laboratory to control the AT and RH, as well as isolate the influence of ambient light—all to overcome the limitations and uncertainties present in the current experiment. In the analysis of droplet area and contact angle, we will use Polymethyl Methacrylate (PMMA) as the non-hydrophilic material for a control experiment. Additionally, we will consider the influence of other factors—such as the droplet’s surface tension—on the irregular shape of the droplet. At the same time, we will also find more biological samples for shooting to expand the application field of this equipment.

## Figures and Tables

**Table 1:** Comparison table of performance parameters for different common-path off-axis DHM.

Device	FOV/μm	Fringe Ratio/%	Speed/fps	Resolution/μm
PSDHM	584 × 438	100	34.8	2.76
LSDHM [8]	116 × 116	100	30	/
CPDHM [25]	189 × 225	100	90	/
DHM based on Multiple Biprisms/Biprism [15]	234 × 175	73.7/41.7 (Biprism)	10	2.19

## Data Availability

Data are contained within the article.

## Abbreviations

The following abbreviations are used in this manuscript:

DHM	digital holographic microscopy
PSDHM	prism-based self-referencing digital holographic microscopy
OPD	optical path difference
BS	beam splitter
PBS	polarization beam splitter
FOV	field of view
RH	relative humidity
AT	temperature
CCR	constant contact radius
SS	stick-slip
SJ	stick-jump
PMMA	polymethyl methacrylate
